# Supplementary material for: A Combinatorial Single-Molecule Real-Time and Illumina Sequencing Analysis of Postembryonic Gene Expression in the Asian Citrus Psyllid Diaphorina citri
Source: Insects. 2024 May 28;15(6):391. doi: 10.3390/insects15060391 (PMC11203772; doi:10.3390/insects15060391)

Figure S2. CIRCOS visualization of gene and transcript density compared PacBio SMRT sequences with *D. citri* reference genome for eight top lengths of scaffolds. A: Eight longest scaffolds schematic; B: Heat map of gene density distribution in the genome; C: Heat map of gene density distribution of PacBio SMRT sequences; D: Heat map of transcript density distribution in the genome; E: Heat map of transcript density distribution of PacBio SMRT sequences; F: Heat map of LncRNA density distribution on chromosomes.

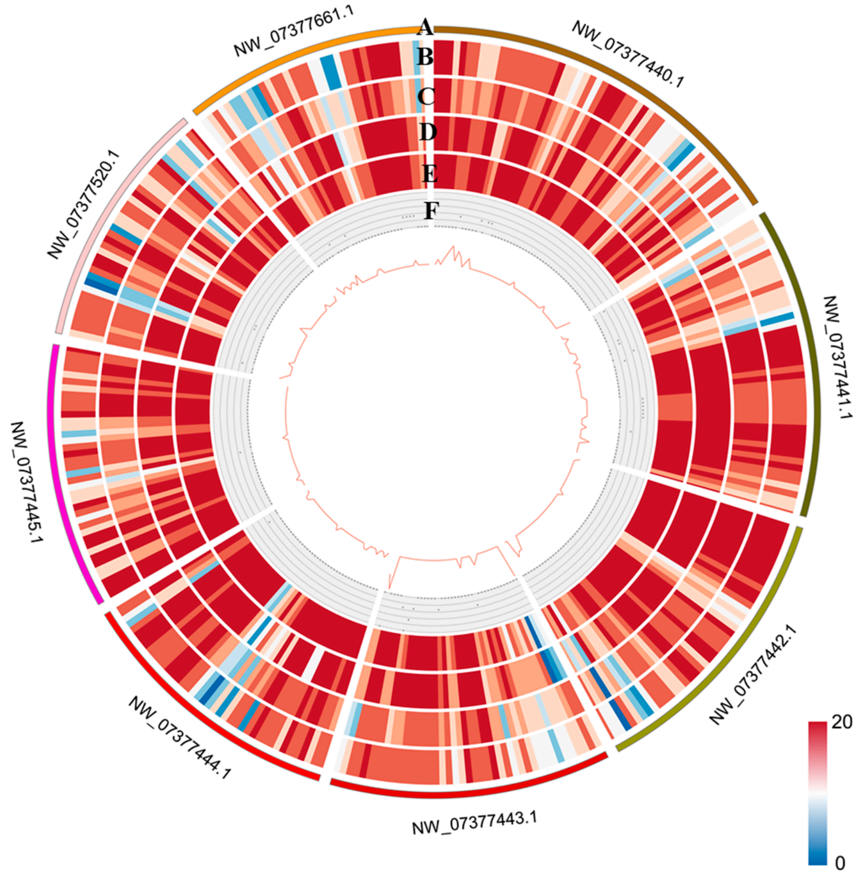

Supplement: Supplementary file 1 [file insects-15-00391-s001.zip › Figure S2.pdf]
