# Supplementary material for: A Combinatorial Single-Molecule Real-Time and Illumina Sequencing Analysis of Postembryonic Gene Expression in the Asian Citrus Psyllid Diaphorina citri
Source: Insects. 2024 May 28;15(6):391. doi: 10.3390/insects15060391 (PMC11203772; doi:10.3390/insects15060391)

Figure S3. Gene Ontology (GO) annotation of DEGs. Red represents biological process; green represents cellular component; and blue represents molecular function. The x-axis represents GO categories; the y-axis (left) represents the percentage of genes; and the y-axis (right) represents the number of genes.

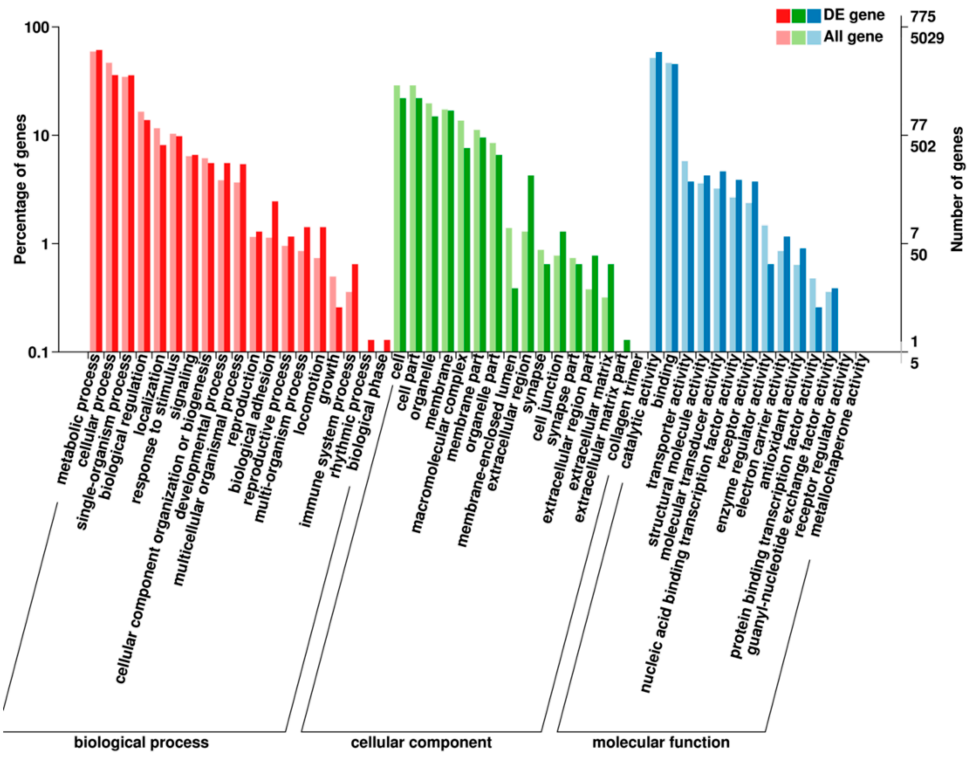

Supplement: Supplementary file 1 [file insects-15-00391-s001.zip › Figure S3.pdf]
