# Supplementary material for: A Combinatorial Single-Molecule Real-Time and Illumina Sequencing Analysis of Postembryonic Gene Expression in the Asian Citrus Psyllid Diaphorina citri
Source: Insects. 2024 May 28;15(6):391. doi: 10.3390/insects15060391 (PMC11203772; doi:10.3390/insects15060391)

Figure S4. Kyoto Encyclopedia of Genes and Genomes (KEGG) annotation of DEGs. Yellow column represents the DEGs involved in cellular process; the purple column represents the DEGs involved in environmental information processing, the pink column represents the DEGs involved in genetic information processing; and the green column represents the DEGs involved in metabolism.

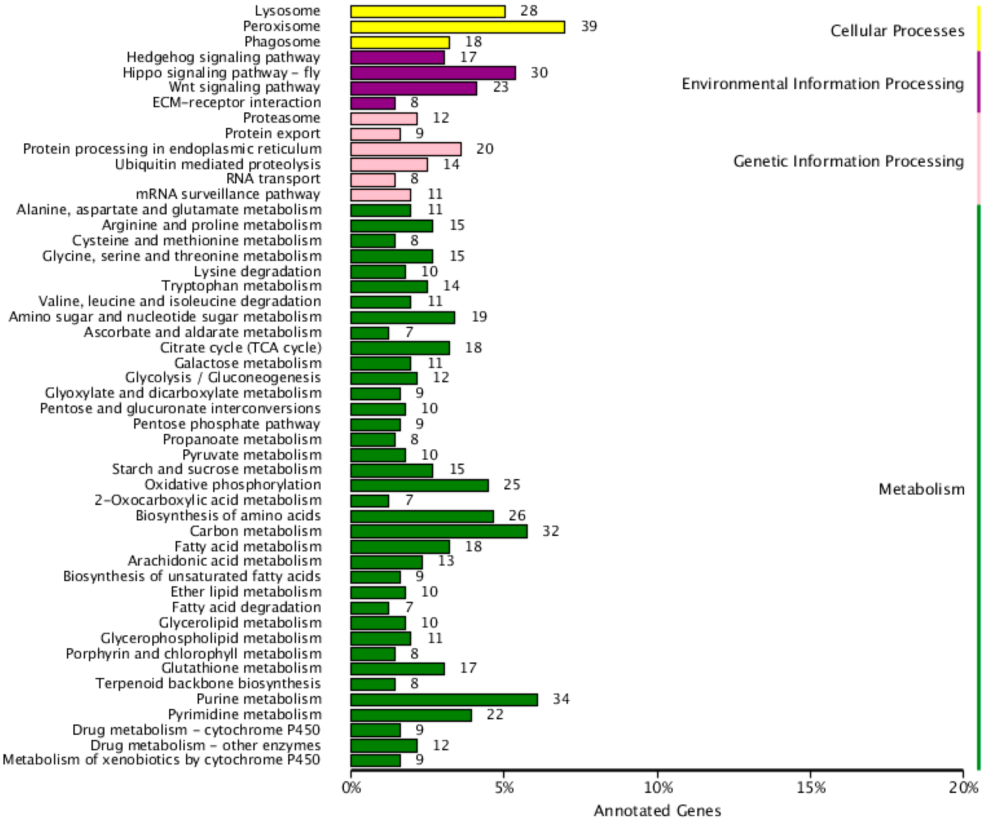

Supplement: Supplementary file 1 [file insects-15-00391-s001.zip › Figure S4.pdf]
