# Supplementary material for: A Combinatorial Single-Molecule Real-Time and Illumina Sequencing Analysis of Postembryonic Gene Expression in the Asian Citrus Psyllid Diaphorina citri
Source: Insects. 2024 May 28;15(6):391. doi: 10.3390/insects15060391 (PMC11203772; doi:10.3390/insects15060391)
Supplement: Supplementary file 1 [file insects-15-00391-s001.zip › Table S2.pdf]

Table S2. Summary of reads by Illumina sequencing of *D. citri*.

Sample name	Reads number	Base number	GC content (%)	Q20 (%)	Q30 (%)
Nymph-1	24,210,396	7,235,076,766	43.54	97.66	93.65
Nymph-2	21,614,043	6,454,230,450	42.34	97.61	93.53
Nymph-3	22,658,114	6,766,436,178	43.66	97.62	93.56
Adult-1	20,546,970	6,129,989,232	41.31	97.83	93.9
Adult-2	21,889,757	6,540,931,744	40.3	97.57	93.29
Adult-3	20,831,850	6,217,810,638	40.38	97.81	93.81
