# Supplementary material for: A Combinatorial Single-Molecule Real-Time and Illumina Sequencing Analysis of Postembryonic Gene Expression in the Asian Citrus Psyllid Diaphorina citri
Source: Insects. 2024 May 28;15(6):391. doi: 10.3390/insects15060391 (PMC11203772; doi:10.3390/insects15060391)
Supplement: Supplementary file 1 [file insects-15-00391-s001.zip › Table S3.pdf]

Table S3. Annotation of new transcripts of *D. citri*.

Annotation databases	Number of new transcripts	Percentage of new transcripts
COG	2391	36.85%
GO	2894	44.60%
KEGG	2998	46.20%
KOG	4175	64.34%
Pfam	4616	71.14%
Swiss-Prot	3705	57.10%
eggNOG	5285	81.45%
Nr	6451	99.41%
All	6489	-
