# Supplementary material for: A Combinatorial Single-Molecule Real-Time and Illumina Sequencing Analysis of Postembryonic Gene Expression in the Asian Citrus Psyllid Diaphorina citri
Source: Insects. 2024 May 28;15(6):391. doi: 10.3390/insects15060391 (PMC11203772; doi:10.3390/insects15060391)
Supplement: Supplementary file 1 [file insects-15-00391-s001.zip › Table S4.pdf]

Table S4. Statistical analysis of SSRs number.

Searching item	Numbers
Total number of sequences examined	8,553
Total size of examined sequences (bp)	17,365,293
Total number of identified SSRs	5,061
Number of SSR containing sequences	3,267
Number of sequences containing more than 1 SSR	1,188
Number of SSRs present in compound formation	539
Mono-nucleotide	4,138
Di-nucleotide	325
Tri-nucleotide	527
Tetra-nucleotide	65
Penta-nucleotide	4
Hexa-nucleotide	2
