# Supplementary figures and images for: A Combinatorial Single-Molecule Real-Time and Illumina Sequencing Analysis of Postembryonic Gene Expression in the Asian Citrus Psyllid Diaphorina citri
Source: Insects. 2024 May 28;15(6):391. doi: 10.3390/insects15060391 (PMC11203772; doi:10.3390/insects15060391)

Figure S1. BUSCO assessment results in this study.

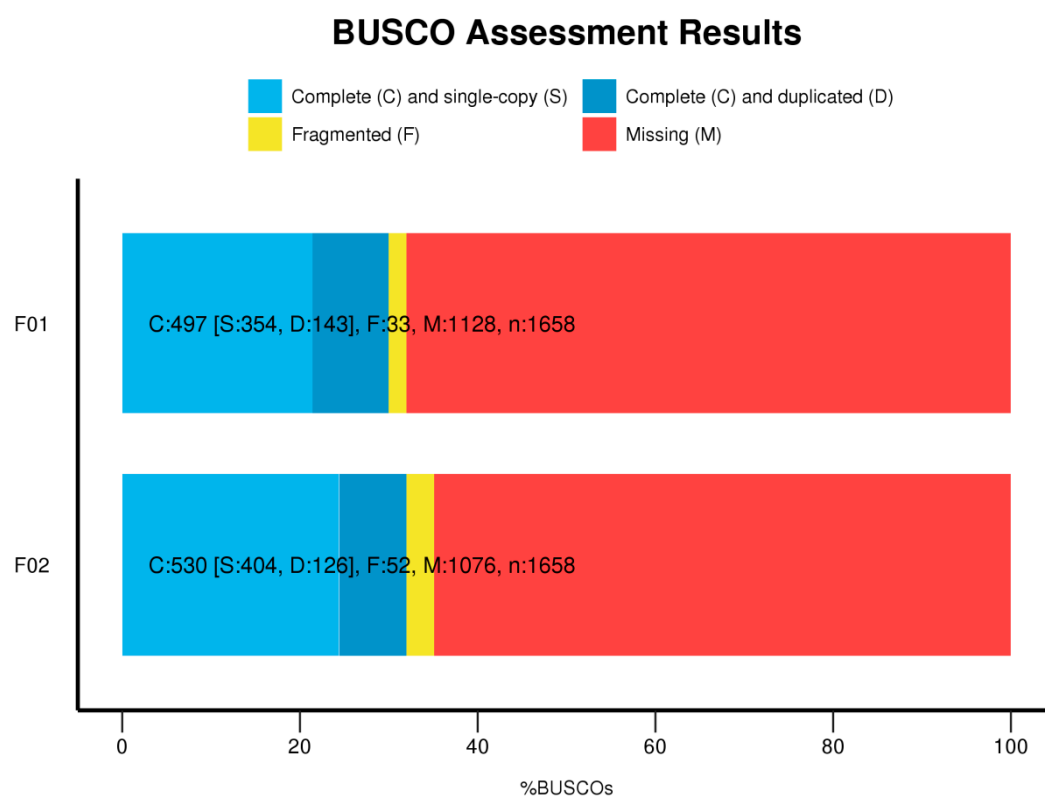

Supplement: Supplementary file 1 [file insects-15-00391-s001.zip › Figure S1.pdf]
